# Morphometric Analysis of Hepatic Steatosis in Chronic Hepatitis C Infection

**DOI:** 10.4103/1319-3767.45047

**Published:** 2009-01

**Authors:** Alia Zubair, Shahid Jamal, Azhar Mubarik

**Affiliations:** Department of Histopathology, Army Medical College, Rawalpindi, Pakistan

**Keywords:** Chronic hepatitis C, fibrosis, morphometry, steatosis

## Abstract

**Background/Aims:**

To quantitatively assess steatosis by a morphometric method and to study its relationship with other histological features of chronic hepatitis C (CHC). This was a comparative descriptive study. The study was carried out in the Department of Histopathology, Army Medical College, Rawalpindi, Pakistan, from March 2006 to March 2007.

**Methods:**

Patients who had undergone a liver biopsy for the evaluation of hepatitis C virus (HCV) infection were included in the study. Demographic characteristics and laboratory data were collected at the time of biopsy. The first hundred biopsy specimens that met the inclusion criteria were assessed for grades of steatosis (semiquantitatively), diameter of fat globules (by a morphometric method), necroinflammation, and fibrosis (semiquantitatively). Liver biopsies were processed for paraffin embedding, stained with hematoxylin and eosin, whereas Gomori's Reticulin stain was used for the evaluation of fibrosis.

**Results:**

Out of 46 cases showing fatty change, pansteatosis was observed in 24 (52%) patients: 12 (26%) cases had a pericentral and mid zonal distribution of fat globules and eight (17.5%) cases revealed a mid zonal pattern only. There were two (4.5%) cases in which fat globules were found in periportal and mid zonal areas. None of the histological parameters (the stage of fibrosis and grades of inflammation) had any significant correlation with these distribution patterns of steatosis. The diameter of fat droplets was quantified by morphometry. A mixed pattern of steatosis was observed more frequently (21 out of 46 cases): 17 cases had microglobules and eight biopsies showed macroglobules. The size of the fat globules exhibited a significant correlation with the stage of fibrosis (*P* < 0.0001). The analysis of the grades of necroinflammation did not reveal any significant relationship with the diameter of fat globules.

**Conclusions:**

A mixed pattern of fat globules is more frequently observed in CHC, but macrovesicular steatosis is associated with a higher stage of fibrosis. Morphometry is recommended as one of the important tools for the follow-up of HCV-infected patients. Whether an accurate assessment of fat globule size by morphometry is preferred for the evaluation of patients before and after the antiviral therapy needs further research.

The burden of hepatitis C virus (HCV)-related chronic liver disease (CLD) in Pakistan has increased. Earlier studies showed that of all patients presenting with CLD, 16.6% were positive for anti-HCV antibodies.[1] More recent data shows that nearly 60–70% patients with CLD tend to be positive for anti-HCV antibodies.[2]

Steatosis is a common histological feature of chronic hepatitis C (CHC). The overall prevalence of steatosis in CHC patients is around 55% (range: 32.8–81.2%)[3] in Western countries and 61.5–65.5% in Pakistan.[4,5] This complication of CHC may have implications for its long-term prognosis.[3] Hepatologists and gastroenterologists rely on the histological findings to determine the nature and extent of the hepatic damage and to select an appropriate treatment course for chronic HCV infection.[6] Conventional routine histological examination is the most common method, although it is subjective and not quantitative.

It is not yet clear as to what extent the fat accumulation contributes to the liver damage. There is no study available in Pakistan in which the size and distribution of fat globules have been determined. Studies carried out in the Western countries have assessed the amount of fat in the liver by semiquantitative methods[7] or quantitatively by using automated computerized procedures.[8] The quantitative method is thought to be more reproducible than the semiquantitative grading of steatosis.

There is a need to accurately assess the quantity of steatosis in liver biopsies for the pathological, clinical, and prognostic evaluation of CHC patients. Thus, the present study was designed to assess the fat content in these patients by using a morphometric method to analyze its relationship with other histological features in CHC patients.

## PATIENTS AND METHODS

The study was carried out from March 2006 to March 2007 in the Department of Pathology (Histopathology), Army Medical College, Rawalpindi, Pakistan. Liver biopsies (longer than 1 cm and containing more than five portal tracts) of 100 CHC patients were included in this study. None of the patients had received any antiviral therapy. Patients included in the study had elevated serum alanine aminotransferase levels (1.5-fold above the upper limit of the normal range for more than six months prior to biopsy). Hepatitis C viral infection was confirmed by the presence of anti-HCV antibody as well as HCV RNA (positive on polymerase chain reaction). Patients with clinical evidence of cirrhosis, ascites, variceal bleeding, or any other associated liver disease, were excluded from the study. All patients were nonalcoholic, nondiabetic, nonobese, and nonhypertensive.

Liver biopsies were processed for paraffin embedding and stained with hematoxylin and eosin (H&E), whereas Gomori's Reticulin stain was used for the evaluation of fibrosis. Modified Knodell's scoring system proposed by Ishak *et al*. was used to evaluate separate scores for the degree of necroinflammation (HAI-Histologic activity index) and fibrosis.[9]

The distribution of fat globules was defined as being pericentral, mid-zonal, or periportal when the globules were confined to zones 1, 2, or 3, respectively. Pansteatosis was defined as steatosis involving all zones.[10] The distribution of fat globules was microscopically assessed under 10× objective magnification in all the biopsies showing steatosis.

The steatosis was graded using the method proposed by Brunt (1999), which is based on the percentage of the total hepatocyte volume affected by fat. Three fields per zone (where maximal steatosis was observed) were analyzed under 40× magnification, and the results of each sample were averaged and graded as none, mild (involving < 10% of hepatocytes), moderate (involving 10–30% of hepatocytes), and severe steatosis (involving > 30% of hepatocytes).[7] Morphometry was used to assess the diameter of fat globules in H & E-stained sections of biopsies with steatosis. Morphometry is the measurement of forms, and it is used in histopathology to describe measurements made from two-dimensional sections.[10] As described earlier, ten fields were chosen for morphometric analysis around portal tract areas, central vein areas, and mid zone (three fields from each zone and one randomly selected zone displaying maximum steatosis).[10]

The diameter of a fat globule was measured by using a Nikon ocular micrometer under 100× magnification, and then, the mean diameter was calculated (in micrometers). The level of maximum diameter was identified by focusing up and down through the planes of the section; the diameter at this level was measured with an eyepiece micrometer. The true diameter of each fat globule transected by thin sections was calculated from the mean diameter of the transactions as 4d/∏.[11]

After calculating the diameters, fat globules were graded as described by Zaitoun *et al*.[10]

- Microvesicular steatosis (microglobules): The mean diameter of fat globules plus one standard deviation is less than 15 *μ*m (M + SD < 15 *μ*m).
- Macrovesicular steatosis (macroglobules): The mean diameter of fat globules minus one standard deviation is equal to or larger than 15 *μ*m (M – SD ≥ 15 *μ*m).
- Mixed microvesicular and macrovesiular steatosis: The mean diameter of fat globule plus or minus one standard deviation is more or less than 15 *μ*m (M ± SD ≈ 15 *μ*m).

The data were fed into a computer program Statistical Package for Social Sciences (SPSS) version 15 for Windows, and the correlation of steatosis with grades and stages of fibrosis and necroinflammation was calculated by using Spearman correlation. Results were considered significant if *P* < 0.05.

## RESULTS

Hepatic steatosis was found in 46 out of 100 CHC patients. Pansteatosis was observed in 24 (52%) patients; 12 (26%) cases had pericentral and mid-zonal distribution of fat globules, and eight (17.5%) cases revealed a mid-zonal pattern only. In two (4.5%) cases, fat globules were found in the periportal and mid-zonal areas.

Table 1 presents the correlation of histological parameters in 46 CHC patients with the distribution of fat globules. None of these parameters (stage of fibrosis or grades of inflammation) had shown any statistically significant correlation with the distribution patterns of steatosis.

**Table 1:** Analysis of correlation between patterns of distribution of fat globules and other variables (*n* = 46)

Variables	*r*	*P*
Diameter of fat globule	-0.091	0.549
Stage of fibrosis	-0.152	0.313
Histologic activity index	0.021	0.891
Portal inflammation	0.117	0.440
Periportal hepatitis	0.027	0.857
Bridging necrosis	-0.113	0.454
Focal necrosis	0.164	0.275

The diameter of fat globules was calculated in the pericentral and periportal areas and in the mid zone. The grades of fat globules in 46 CHC patients (with steatosis) revealed that 21 (46%) out of 46 cases had a mixed pattern of steatosis, 17 (37%) cases had microglobules, and eight (17%) cases showed macroglobules.

The size of fat globules exhibited a significant correlation with the stage of fibrosis, *P* < 0.0001 [Table 2]. It was observed that the proportion of cases showing fibrosis stages 2 and 3 was higher in patients with macrosteatosis (6/8) than in those with microsteatosis (11/17) and a mixed pattern (13/21).

**Table 2:** Analysis of correlation between diameters of fat globules and other variables (*n* = 46)

Variables	*r*	*P*
Stage of fibrosis	0.570	<0.0001
HAI	0.108	0.475
Portal inflammation	0.026	0.865
Periportal hepatitis	0.039	0.799
Bridging necrosis	0.228	0.127
Focal necrosis	0.027	0.857

Statistically, no significant correlation was found between the diameter of fat globules and the grades of necroinflammation (*P* = 0.475). Similarly, the analysis of the individual components of inflammation did not reveal any significant relationship with the diameter of fat globules [Table 2].

## DISCUSSION

Semigrading of steatosis has been described by many authors.[12] Fully automated methods have also been described[13] but require special staining for fat and could not determine the size of fat droplets. Therefore, in conjunction with a computerized technique, the use of morphometry in detecting micro- and macrosteatosis will probably save CHC-infected livers that were formerly thought to be too steatotic to be transplanted.[14] In the present study, micrometry was used for assessing the diameter of fat globules.

A mixed pattern of steatosis (46%) was more frequently observed in the studied population. The proportion of microglobules (37%) was greater than that of macroglobules (17%). This observation is supported by the data of a recent study showing a higher percentage of microglobules in CHC infection with nongenotype 3.[15] While quantitatively comparing the size of fat globules in CHC patients and in patients with alcoholic liver disease (ALD), Zaitoun *et al*. found a higher proportion of microglobules in CHC.[10] Microvesicular steatosis implies the presence of mitochondrial dysfunction and oxidative stress caused by the core protein.[16] This suggests that the HCV may be directly inducing steatosis in these patients.[17] The fat globules initially formed in zone 3 coalesce to form large droplets to produce macrosteatosis.

In this study, pansteatosis was observed in most of the cases in contrast to another study in which the incidence of pansteatosis was higher in ALD, whereas periportal and other forms of fat distribution were seen in CHC patients.[10]

The assessment of steatosis in individual locations gives a useful clue to the origin of steatosis in CHC infection. The core protein is present in the cytoplasm of hepatocytes principally located around the central veins and within lobules.[18] We also observed the same in this study; the high frequency of pansteatosis (52%) and pericentral with mid-zonal (26%) distribution of steatosis probably reflect the role of the HCV core protein and its direct effects in the pathogenesis of steatosis, further leading to fibrosis in HCV infection. However, statistically, the distribution patterns of steatosis did not show any correlation with the stage of fibrosis and necroinflammatory grades. This explains that the presence of fat at a particular site in the liver does not correlate with a higher stage of the disease.

Data available from studies of nonalcoholic fatty liver disease (NAFLD) describe the significance of distribution of steatosis and its correlation with fibrosis. Perivenular and subsinusoidal fibrosis seen in hepatic steatosis is the association between steatosis and lipid peroxidation in zone 3 hepatocytes.[18] It may relate to the lower pO_2_ observed in this zone with associated oxidative stress and is likely to be related to perivenular fibrosis.[19]

## CONCLUSIONS

A mixed pattern of fat globules is more frequent in CHC, but macrovesicular steatosis is associated with a higher stage of fibrosis. Hence, all biopsies showing macroglobules deserve close follow-up of the patients.

Whether an accurate assessment of the size of fat globule by morphometry is preferred for the evaluation and follow-up of patients before and after an antiviral therapy warrants further investigation.
